# FK506-binding protein 2 (FKBP13) inhibit Bax-induced apoptosis in *Saccharomyces cerevisiae* (yeast)

**DOI:** 10.1007/s10565-021-09633-w

**Published:** 2021-08-03

**Authors:** Damilare D. Akintade, Bhabatosh Chaudhuri

**Affiliations:** 1 School of Clinical and Applied Sciences, Leeds Beckett University, Leeds, LS1 3HE UK; 2 Leicester School of Pharmacy, De Montfort University, Leicester, LE1 9BH UK

**Keywords:** Human FKBP2, Yeast apoptosis, Human FKBP13, Protein folding and apoptosis, FKBP2 and Bax

## Abstract

FK506-binding protein 2 (FKBP13) is a part of the immunophilin protein family involved in immunoregulation. It is also believed to operate as a factor in membrane cytoskeletal framework and as an ER chaperone. FKBP2 (FKBP13) and FKBP1 (FKBP12), known as immunophilins, are binding proteins for rapamycin and FK506, which are immunosuppressive drugs. It was suggested that immunophilin-like and immunophilin proteins play significant roles in regulating intracellular calcium and protein folding/sorting, acting as molecular chaperones. Within the 15 mammalian FKBPs known, FKBP1 is merely the only one proven to form complexes with rapamycin and FK506 in the cytosol and facilitate their T cells immunosuppressive effects, FKBP2 is a luminal protein of the endoplasmic reticulum (ER) and is reported to take part in protein folding in the ER. However, little is known about FKBP2 link with apoptosis (either as a pro or anti-apoptotic protein). In this study, FKPB2 protein was co-expressed with the pro-apoptotic protein Bax after a yeast-based human hippocampal cDNA library screening. The yeast strain carrying the Bax gene was transformed with an episomal 2-micron plasmid that encodes the HA-tagged FKBP2 gene. The resultant strain would allow co-expression of Bax and FKBP2 in yeast cells. The results presented here show that a protein involved in protein folding can play a role in protecting yeast cell from Bax-induced apoptosis.

**Supplementary Information:**

The online version contains supplementary material available at 10.1007/s10565-021-09633-w.

## Introduction

FKPB13 is an endoplasmic reticulum (ER) chaperone (Tat et al. 2021); the FKBP2 gene encodes FKBP13 (Boon et al. 2009); its molecular function includes transport between trans-proline and cis-proline residues for appropriate folding of the protein in the lumen of the ER (Jeong et al. 2017). FKBP13 has been reported to be upregulated through ER stress (Bush et al. 1995) which is believed to be vital for the maintenance of ER protein homeostasis as part of the unfolded protein response (Jeong et al. 2017). ER stress could mediate the start and advancement of diseases (Kropski and Blackwell 2018). ER stress was linked to inducing apoptosis in alveoli type 2 cells (Katzen et al. 2019) and the differentiation of the myofibroblast and T cell (Kropski and Blackwell 2018). FK506-binding protein 2 (FKBP2) is part of the immunophilin protein family. FKBP2 is involved in immunoregulation; it is also believed to operate as a factor in membrane cytoskeletal framework and ER chaperone (Kim et al. 2017). FK506-binding proteins (FKBPs) and cyclophilins bind to FK506 and cyclosporine A (CsA) to form complexes that inhibit the protein phosphatase calcineurin that regulates T cell proliferation and also has a role in neuronal apoptosis (Nigam et al. 1993). Activation of T cells is prevented by FK506 and CsA, which are immune-suppressants. Because of their indirect association with immune suppression via calcineurin inhibition, FKBPs and cyclophilins are referred to as immunophilins (Nigam et al. 1993). FKBP2 (FKBP13) and FKBP1 (FKBP12), known as immunophilins, are binding proteins for rapamycin and FK506, which are immunosuppressive drugs (Nigam et al. 1993).

The FKBPs and cyclophilins function as cis–trans prolyl isomerases, which have a profound role in protein folding (Edvardsson et al. 2007). FKBP12 (FKBP1) and FKBP13 (FKBP2) are two proteins with comparable molecular weight, 12 and 13 kDa. They share 43% similarity in their amino acid sequences. FKBP1 is cytosolic, whereas FKBP2 is membrane-bound and resides in the ER. FKBP2 possesses a COOH-terminal RTEL motif and an NH2-terminal signal sequence, alleged to take part in ER retention and targeting of the protein to the ER (Walensky et al. 1998). After being retained in the ER, FKBP2 binds to ER membranes with its 21 amino acid membrane-binding domain (Nigam et al. 1993). FKBP2, like the immunoglobulin-binding protein (BiP), is thought to take part in protein folding and act as a molecular chaperone in the lumen of the ER (Padilla et al. 2003; Walensky et al. 1998). It has also been suggested that FKBP2 influences vesicular trafficking (Padilla et al. 2003).

Folding of secreted proteins occurs in the ER. Reactive oxygen species (ROS) levels are elevated when there is a defect in protein folding within the ER; these elevated ROS levels can lead to defective electron transport chains causing hyper-activation of nicotinamide adenine dinucleotide phosphate H (NADPH) oxidase within the ER culminating in the triggering of programmed cell death (Akintade and Chaudhuri 2020c; Haynes et al. 2004; Leadsham et al. 2013). It can be speculated that, like binding immunoglobulin protein (BiP), FKBP2 protects the ER from ROS damage. FKBP2 may also play a part outside the ER, such as in cytoskeletal support of membranes (Walensky et al. 1998). FK506, CsA and rapamycin, another immune-suppressant, prevent the growth of some strains of the yeast *Saccharomyces cerevisiae* (Nielsen et al. 1992). Yeast also has FK506-binding proteins, yFKBP12 and yFKBP13. Yeast strains lacking yFKBP12 are resistant to rapamycin but are sensitive to FK506 (Nielsen et al. 1992). Immense rates of secretion and synthesis of immunoglobulin (Ig) exposes plasma cells to huge endoplasmic reticulum (ER) stress (Jeong et al. 2017). FK506-binding protein 13 protein has been linked to quality control that protects plasma cells from ER stress, which could trigger an associated apoptosis response (Jeong et al. 2017). Protein homeostasis is vital for the existence of plasma cells; while the molecular mechanism is generally unknown, FKBP2 protein overexpression was reported to be present in long-lived plasma cells from autoimmune mice (Jeong et al. 2017). This shows the need for an understanding of protein homeostasis.

Redox homeostasis is maintained by cells using stored antioxidant enzymes to mend oxidative damage and neutralise excessive ROS levels produced by organelles within cells and external sources (Perrone et al. 2008). If the concentrations of internal ROS continue to increase above a specific limit, it would invariably lead to oxidative stress leading to the accumulation of oxidised proteins, DNA, and lipids (Akintade and Chaudhuri 2020b; Drakulic et al. 2005). FKPB2 has not directly been linked to apoptosis. Higher FKBP13 protein expression has been seen in several fibrotic lung diseases (Li et al. 2021). FKBP13 knock out mice was reported to have amplified lung inflammation and heightened predisposition to alveoli type 2 cells apoptosis (Li et al. 2021). Lack of FKBP13 blocked late-stage fibrosis resolution because of increased apoptosis of lung epithelial cells induced by ER stress (Li et al 2021). However, according to Bush et al. (1994), the ER’s lumen is the initial site for correct folding and assembly of secretory proteins and ER-bound trans-membrane proteins. FKPB2 could be considered a molecular chaperone, which binds briefly to proteins that have been translocated into the ER and facilitated their folding. FKBP2 gene has been linked to obsessive osteoporosis (Kim et al. 2017) and type 2 diabetes (Lu et al. 2008). FKBP2 was also reported to be expressed in tissues that are susceptible to hyperplasia in MEN1 (multiple endocrine neoplasia type-1) patients; nonetheless, FKBP2 was excluded in the mutation analysis of MEN1 tumours as a possible biomarker gene for MEN1 (Kim et al. 2017). The anti-apoptotic property of FKBP2 was studied in this research along with Bcl-xL, a Bcl-2 protein family, which is anti-apoptotic. FKBPs are a distinct group of chaperones located in a broad diversity of organisms (Somarelli et al. 2008). Some of the cellular function they carry out include apoptosis modulation, protein folding, assembly of histone, cytokines regulation, binding to nucleic acid, and steroid receptor complexes transport (Somarelli et al. 2008). Several of these roles involve certain domains which adjust to definite tertiary forms. Humans, FKBP2 have adjustable amounts of FKBP domains which are linked with the ER (Somarelli et al. 2008).

Some novel genes were discovered to salvage Bax-induced apoptosis in screening the human hippocampal cDNA library in yeast, as patented and described previously (Publication number: 20090258794) (Chaudhuri 2009). FKBP2 (a gene involved in protein folding) gene was one of the genes identified and has been chosen for further investigation in this study to corroborate its role in apoptosis inhibition. Yeast strain carrying one copy of integrated Bax gene leu2::LEU2-Bax was transfected with episomal (2-micron) plasmid, which encodes FKBP2 and Bcl-xL genes (HA-tagged) (supplementary information). The resultant strains leu2::LEU2-Bax pSYE/FKBP2-HA allows the co-expression of Bax and FKBP2 in the yeast strain, while leu2::LEU2-Bax pSYE/Bcl-xL-HA allows the co-expression of Bax and Bcl-xL in yeast strain. The negative control strain is leu2::LEU2-Bax pSYE-HA.

## Materials and methods

### Yeast strains

The yeast strain W303-1A Mat*a (MATa ade2-1 ura3-1 his3-11 trp1-1 leu2-3 leu2-112 can1-100)* (ATCC #208,352) is auxotrophic for *ADE2*, *HIS3*, *LEU2*, *TRP1* and *URA3*. New yeast strains were created by transforming integrative plasmids (Supporting Information, Sects. 1, 2, 3), which would express Bax from the GAL1 promoter or episomal plasmid expression FKBP2 or Bcl-xL gene on PGK1 promoter.

### Yeast transformation

Plasmid bearing Bax gene expression cassettes under the control of the galactose-inducible *GAL1* promoter (*GAL1*p; see Supporting Information, Sects. 1) was used for genomic integration at the *LEU2* chromosomal loci of the yeast strain to yield strains that contain one copy of Bax,—leading to the generation of leu2::LEU2-Bax yeast strain. And an episomal plasmid bearing FKBP2 or Bcl-xL gene expression cassettes on a PGK1 promoter, creating leu2::LEU2-Bax pSYE/FKBP2-HA, leu2::LEU2-Bax pSYE/Bcl-xL-HA, and leu2::LEU2-Bax pSYE-HA (negative control) yeast strains. The transformation was carried out using a published protocol (Kawai et al. 2010).

### Detection of dead cells with phloxine B dye

Determining cell viability is one of the most common methods for measuring the impact of different types of irritants or stressors in toxicity studies. Viability shows the proportion of live cells in the entire populace. Phloxine B (a red dye) is water-soluble and could be used to stain dead cells of many cells, including yeasts (*Saccharomyces cerevisiae*). Cell death was assessed by staining cells with the red dye phloxine B (Sigma, P-4030-25G) (Kwolek-Mirek and Zadrag-Tecza 2014). Live cells expel the dye, whereas it is accumulated in dead cells. This can be observed by fluorescence microscopy. Staining experiments were performed exactly as published earlier (Derf et al. 2018).

### Detection of ROS with dihydroethidium

ROS are biological products from the normal metabolism of oxygen. ROS plays an essential role in cell signalling. Nonetheless, ROS levels could increase significantly throughout oxidative stress and accumulate, resulting in significant damage to the cell. Fluorimetric ROS assay kit uses a unique ROS sensor to quantify ROS in cells. AAT Bioquest Fluorimetric Intracellular Total ROS Activity Assay Kit (#22,901) was used for measuring ROS. Experiments were performed as published earlier (Derf et al. 2018).

### Quantifying mitochondrial membrane potential with the JC-10 dye

Mitochondria are the critical powerhouse of cells, and it plays crucial roles in cell proliferation and apoptosis. Mitochondrial membrane potential assay kit detects the mitochondrial membrane potential in living cells. AAT Bioquest JC-10 mitochondrial membrane potential assay kit (#22,800) uses water-soluble JC-10 to determine MMP quantitively. Experiments were conducted as per the published protocol (Derf et al. 2018).

### Staining with Hoechst dye for monitoring live cells

Hoechst 33,258 (Thermo Fisher Scientific; #H21491) is a nucleic acid stain widely used to detect live cells. When bound to double-stranded DNA, the dye emits blue fluorescence. Staining with the dye was performed as described earlier (Derf et al. 2018).

### Assessing nuclear DNA fragmentation via the TUNEL assay

AAT Bioquest TUNEL apoptosis assay kit (#22,844) was used to detect nuclear DNA fragmentation (NDF). The assays were performed as described earlier (Derf et al. 2018).

### Western blotting

Western blotting was carried out using standard protocols (Von Der Haar 2007), using primary antibodies specific to c-Myc (Thermo Scientific, #MA 1–980) and HA-tag (Proteintech, #51,064–2-AP) or β-actin (Proteintech; #60,008–1-Ig).

## Results and discussion

### Co-expression of Bax on GAL1 promoter with FKBP2 and Bcl-xL on PGK1 promoter

**Fig. 1:**
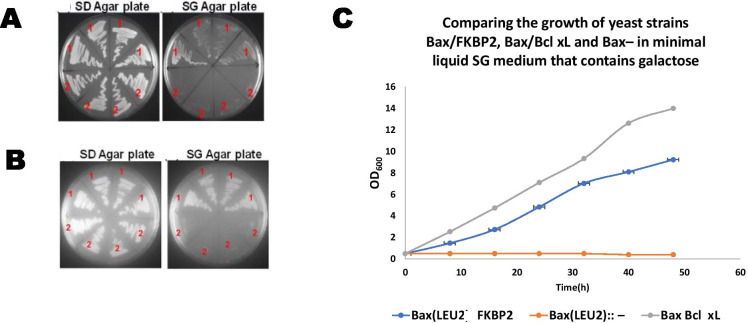
**A** and **B** Growth of yeast strains in solid agar plates over 72 h, in glucose-containing minimal medium (SD), and galactose-containing minimal medium (SG). The 4 transformants on the upper half of the two plates are leu2::LEU2-Bax pSYE/FKBP2-HA **(A)** and leu2::LEU2-Bax pSYE/Bcl-xL-HA **(B)** transformants **(1)**, containing an episomal 2µ-plasmid plasmid that encodes the HA-tagged FKBP2 **(A)** and Bcl-xL **(B)** genes and a Bax expression cassette integrated at the LEU2 locus. The 4 transformants, in the lower half of the plates **(A)** and **(B)**, is leu2::LEU2-Bax pSYE-HA **(2)** transformants which contain the Bax expression cassette and an empty plasmid. **C** Growth of yeast cells, leu2::LEU2-Bax pSYE/FKBP2-HA and leu2::LEU2-Bax pSYE/Bcl-xL-HA, in minimal liquid medium containing galactose, throughout 48 h along with negative control strain. A two-tailed paired sample t-test show, statistically, that there was a significant difference (*p* < 0.05) compared to the control, but the difference between FKPB2 and Bcl-xL is not significant (*p* > 0.05)

**Fig. 2:**
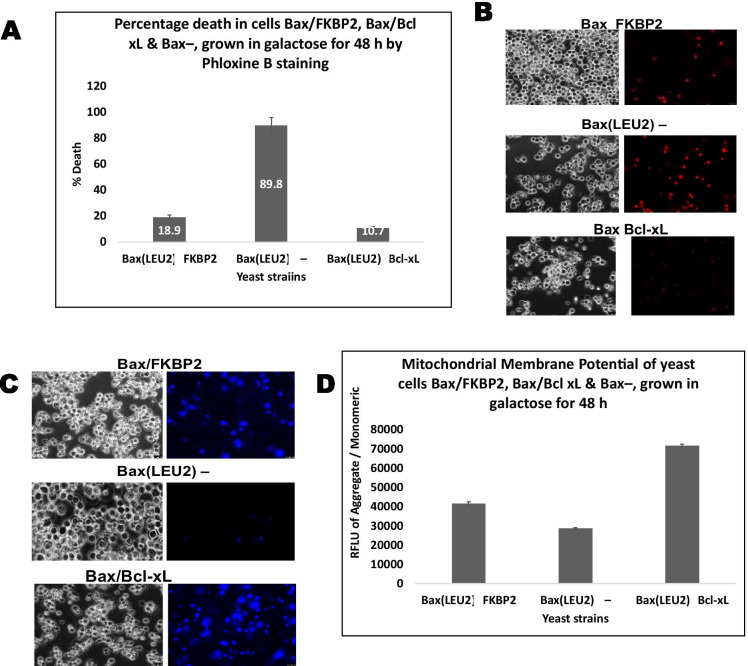
**A** The percentage cell death in strains leu2::LEU2-Bax pSYE/FKBP2-HA, leu2::LEU2-Bax pSYE/Bcl-xL-HA and control leu2::LEU2-Bax pSYE-HA, after growth in galactose for 48 h. **B** Dead cells stained with phloxine B are shown in. A two-tailed paired sample *t*-test show, statistically, that there was a significant difference (*p* < 0.05) compared to the control, but the difference between FKPB2 and Bcl-xL is not significant (*p* > 0.05). **(C)** Visualisation of yeast cells that co-express Bax and FKBP2 and Bcl-xL or Bax alone, after staining with Hoechst 33,342 dye. **D** Quantification of the mitochondrial membrane potential of yeast strains, leu2::LEU2-Bax pSYE/FKBP2-HA which co-expresses Bax and FKBP2**,** leu2::LEU2-Bax pSYE/Bcl-xL-HA which co-expresses Bax and Bcl-xL, and leu2::LEU2-Bax pSYE-HA which expresses Bax alone, using a fluorescent plate reader. A two-tailed paired sample *t*-test shows that there was a significant difference (*p* < 0.05) compared to the control, but the difference between FKPB2 and Bcl-xL is not significant (*p* > 0.05)

**Fig. 3:**
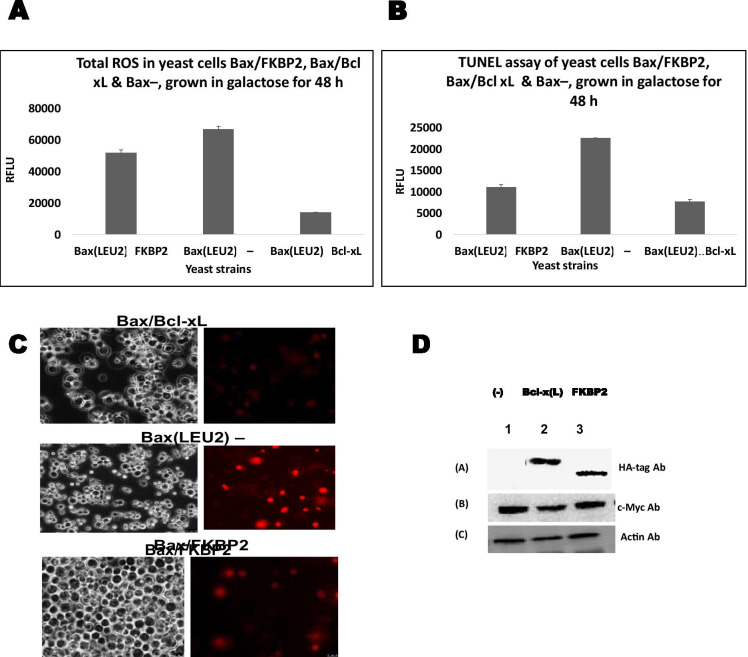
**A** Measurement of ROS produced in the yeast strains leu2::LEU2-Bax pSYE/FKBP2-HA, leu2::LEU2-Bax pSYE/Bcl-xL-HA and leu2::LEU2-Bax pSYE-HA, A two-tailed paired sample *t*-test show that there was a significant difference (*p* < 0.05) compared to the control, but the difference between FKPB2 and Bcl-xL was not significant (*p* > 0.05). **B** TUNEL assay comparing the nuclear DNA fragmentations in yeast strains leu2::LEU2-Bax pSYE/FKBP2-HA, leu2::LEU2-Bax pSYE/Bcl-xL-HA and leu2::LEU2-Bax pSYE-HA. The cells FKBP and Bcl-xL have lower nuclear DNA fragmentation compared to leu2::LEU2-Bax pSYE-HAstrain. A two-tailed paired sample t-test show that there was a significant difference (*p* < 0.05) compared to the control, but the difference between FKPB2 and Bcl-xL is not significant (*p* > 0.05). **C** Represent the microscopic image of cells after tunel assay, leu2::LEU2-Bax pSYE/FKBP2-HAand leu2::LEU2-Bax pSYE/Bcl-xL-HA. **D** Western blot to monitor the presence of Bax in yeast cells that express only Bax and in cells that co-express Bax together with FKBP2 and, Bcl-xL. A 10 µg of total cellular proteins in cell lysates were loaded onto each lane, and the blot was probed with an antibody that recognises the c-myc-tag, Actin, and HA-tag

In this study, FKPB2 protein was co-expressed with the pro-apoptotic protein Bax. The yeast strain carrying the Bax gene (leu2::LEU2-Bax) was transformed with an episomal 2-micron plasmid that encodes the HA-tagged FKBP2 gene (see supplementary information). The resultant strain leu2::LEU2-Bax pSYE/FKBP2-HA would allow co-expression of Bax and FKBP2 in yeast cells. Bax is under the control of the GAL1 promoter, while the PGK1 promoter drives FKBP2 expression. Figure 1 show the growth assays (solid and liquid media) of the leu2::LEU2-Bax pSYE/FKBP2-HA strain along with positive (leu2::LEU2-Bax pSYE/Bcl-xL-HA) and negative (leu2::LEU2-Bax pSYE-HA) controls. The positive control contains the Bcl-xL gene, a known anti-apoptotic gene, while the negative control has no gene insertion (just the vector). Figure 1 shows that the yeast strains containing pSYE/FKBP2-HA and pSYE/Bcl-xL-HA along with the Bax gene grew in both media, unlike the yeast strain containing the negative control vector gene, which did not grow. Further assays were carried out as shown in Fig. 2 and Fig. 3 to measure apoptosis hallmark in the respective strains.

Phloxin B assay (death assay) shown in Fig. 2 (A and B) measured the percentage of cell death after Bax protein expression with either FKBP2 and Bcl-xL compared with the strain containing the vector gene (negative control). Phloxin B dye stains dead cells while live cells expel the dye; the negative control has significantly (*p* < 0.05) more death than cells containing FKPB2 and Bcl–xL genes. However, the difference between FKPB2 and Bcl-xL is not significant (*p* > 0.05). This result suggests that FKBP2, similar to Bcl-xL (positive control) rescues Bax-induced apoptosis. This result corroborates the growth assay in Fig. 1, which show cell growth in the yeast strains containing pSYE/FKBP2-HA and pSYE/Bcl-xL-HA but no growth in yeast cells containing leu2::LEU2-Bax pSYE-HA. After the growth assay and death assay results, a live assay (Fig. 2C) was performed along with mitochondrial membrane potential assay (MMP) (Fig. 2D). Hoechst 33,342 dye stains live cells (Fig. 2C), the negative control has the least live cells compared to FKBP2 and Bcl-xL. Figure 2D shows the measurement of MMP; again, yeast cells contain FKBP2 and Bcl-xL have higher MMP than the negative control strain. The mitochondrial is the cell’s powerhouse; it produces adenosine triphosphate (ATP). When the mitochondrial is damaged, it could trigger cell death via apoptosis; essentially, mitochondria membrane potential (MMP) is needed for ATP production (Zorova et al. 2018). Decrease in MMP amount to ATP reduction. When a cell reaches a point of no return, the cell loses its MMP and become committed to death. A prolonged plunge or increase of MMP standard levels could provoke an undesirable decline in cell viability and could result in different pathological conditions. The mitochondria is crucial for maintaining cellular viability and health.

Figure 3 show the ROS and TUNNEL assays and western blotting of the proteins. The ROS produced by the strains was measured; the negative control produced the higher ROS, followed by the strain with FKBP2 and then the positive control (Fig. 3A). If ROS level is very high, it could destroy proteins, membranes, nucleic acids, organelles, and lipids; this could, in turn, trigger the activation of apoptosis (programmed cell death) processes (Ghosh et al. 2018). TUNNEL assay (Fig. 3B and C) measures DNA fragmentation, one of the hallmarks of apoptosis. The strain with empty plasmid has the highest DNA fragmentation than the strain with FKBP2 and Bcl-xL; this suggests the rescue of yeast cells from Bax-induced apoptosis. The western blot shows the presence of the respective protein after their expression.

In this study, the expression of FKBP2 protein was shown to rescue Bax-induced apoptosis. FKBP2 expression was reported to be generated mainly in the endoplasmic reticulum lumen after treatment with an ER stressor (i.e. tunicamycin) (Jeong et al. 2017). Also, suppressing of FKBP2 expression was said to have led to an induction of molecules involved in the terminal unfolded protein response (UPR) and ER stress–linked apoptosis. FKBP2 could lower ER stress, but FKBP2 knockdown had a differing effect (Jeong et al. 2017). FKBPs (FK506-binding proteins) are a group of enzymes that has peptidyl-prolyl cis–trans isomerase (PPIase or rotamase) activities (Harrar et al. 2001). Besides the enzymatic activity, the PPIase domain has a hydrophobic core with a drug-binding compact that allows FKBP to operate as an immunophilin (Jeong et al. 2017). Within the 15 mammalian FKBPs known, FKBP12, a typical member is merely the only one proven to form complexes with rapamycin and FK506 in the cytosol and facilitate their T cells immunosuppressive effects (Harding et al 1989; Schreiber 1991). These complexes (rapamycin–FKBP12 and FK506–FKBP12) mainly inhibit the mammalian target of rapamycin (mTOR) and calcineurin (Jeong et al. 2017).

FKBP2 (FKBP13), a known immunophilin, is binding proteins for rapamycin and FK506, which are immunosuppressive drugs. The cells co-expressing FKBP2 with Bax grew on galactose-containing SG agar plates (Fig. 1A), likewise cells co-expressing Bcl-xL with Bax (Fig. 1B), but the control strain did not. FKBP2, an ER luminal protein, was able to rescue Bax-induced apoptosis. The growth curve (Fig. 1C) and death assay (Fig. 2A) corroborated that FKBP2 can rescue Bax-induced apoptosis in yeast. Figure 1 shows the growth of yeast cells carrying FKBP2 in the presence of full-blown Bax expression. All immunophilins possess cis–trans prolyl isomerase activity. The cis–trans prolyl isomerases play a fundamental role in the correct folding of proteins. Yeast FKBP2 (yFKBP2) is 64% similar in protein sequence to human FKBP2 (hFKBP2), whereas yFKBP1 is 51% similar to hFKBP1 (Nielsen et al. 1992). FKBP2 is a luminal protein of the ER and is reported to take part in protein folding in the ER (Bush et al. 1994). FKBPs are a distinct group of chaperones located in a broad diversity of organisms (Somarelli et al. 2008). Some of the cellular function they carry out include apoptosis modulation, protein folding, assembly of histone, cytokines regulation, binding to nucleic acid, and steroid receptor complexes transport (Somarelli et al. 2008). Several of these roles involve certain domains which adjust to definite tertiary forms. Humans, FKBP2 have adjustable amounts of FKBP domains which are linked with the ER (Somarelli et al. 2008). FK506-binding protein 13 (FKBP13) has approximately 43 nucleotides and 51% amino acid sequence homology with FKBP12 (Jin et al. 1991). The drug-binding site amino acid residues of FKBP12 is conserved in FKBP13 (Jin et al. 1991). However, FK506–FKBP13 complex was not found (in vitro) to substantially inhibit calcineurin (Dumont 2000), and no known function of a rapamycin–FKBP13 complex was reported (Jeong et al. 2017). FKBP13 has been said to be located in the ER lumen in the canine's pancreatic cells and stimulated by ER stressors (Bush et al. 1994; Nigam et al. 1993).

Staining with phloxine B and Hoechst dye showed the extent of death and life in the cells represented in Fig. 2A, B, and C, respectively. The figure shows death of cells due to Bax expression and life resulting from FKBP2’s rescuing cells. A similar observation was also recorded for Bcl-xL. Significantly, less cell death occurred in the strain Bax(LEU2)FKBP2 compared to the strain Bax(LEU2)—, which expressed Bax alone, but the difference between Bax(LEU2)FKBP2 and Bax(LEU2)Bcl-xL is not significant. FKBP2 has been recognised in coarse microsomal sub-cellular portions and is more concentrated in samples holding luminal proteins of the ER (Walensky et al. 1998). Expression of FKBP2 is upregulated in response to heat shock and during accrual of unfolded/aggregated proteins in the ER (Walensky et al. 1998). ER stress can easily activate unfolded protein response (UPR) as an adaptive tactic to maintain viability in the process of re-establishing the homeostasis of protein homeostasis (Todd et al. 2008). Nevertheless, in the presence of excessive ER stress outside the capability of UPR adaptive strategy, terminal UPR is then initiated, and apoptosis is executed (Jeong et al. 2017). Furthermore, expression of a DNA damage-inducible transcript 3 (C/EBP homologous protein (CHOP)), which is a pro-apoptotic transcription factor, is a typical indicator for terminal UPR preceding apoptosis (Marciniak et al. 2004). Thus, there might be a link between ER stress-mediated apoptosis and secretory load increase (Puthalakath et al. 2007).

The difference in ROS production between the strains is significant. The strain expressing Bax alone produces far more ROS than the strains co-expressed FKBP2 and Bcl-xL (Fig. 3A). TUNEL assay (Fig. 3B and  C), ROS measurement (Fig. 3A) and mitochondrial potential quantification (Fig. 2D) indicate that FKBP2 can rescue cells from Bax’s toxicity. Figure 3D shows Western blots to confirm the co-expression of FKBP2 and Bax and shows that the proteins were expressed. This is probably the first time that FKBP2 has been linked to apoptosis as a Bax inhibitory protein. It has been suggested that FKBP2 may function as ER molecular support, catalysing the gathering and/or folding of proteins in the ER (Walensky et al. 1998). This function could also involve correcting the folding of misfolded proteins or preventing misfolding of proteins. Bax that delocalised from the cytosol to the mitochondria to exert its toxic effects may do so in a conformational form that is toxic for the cells (Akintade and Chaudhuri 2020a). It may be that FKBP2 somehow prevents this toxic conformation of Bax from being formed.

X-box-binding protein 1 (XBP1) is one of the three branches of the UPR. XBP1 primarily serves to stimulate the enlargement of the ER (Iwakoshi et al. 2003). Because it functions as a transcription factor to generate molecular chaperones that improve ER-associated degradation (ERAD) and protein folding capacity when induced (Jeong et al. 2017). In the event of aggregation, it enables detection by ubiquitin ligases, which results in the delivery of the substrates ubiquitinated to the proteasome (Shiber and Ravid 2014). This suggests that the chaperones perform a vital role in the clearance by the ubiquitin–proteasome system (UPS) of the incurably misfolded proteins. The exact role of FKBP13 in anti-apoptotic activity is unknown. In this study, the anti-apoptotic function of FKBP13 was investigated through Bax-induced apoptosis in the yeast cell. The result shows that FKBP13 could play a crucial role in preventing Bax-induced apoptosis in yeast, which may play a part in the continuous survival of cells and disease development, progression, and treatment.

## Conclusions

In this study, specific trends in the yeast model of FKBP2 ability to rescue Bax's inherent toxicity were observed. These results suggest the involvement of FKBP2 in apoptosis. FKBP2 has not been directly implicated in apoptosis (either as a pro or anti-apoptotic protein) before; the findings presented here show that a protein involved in protein folding can play a role in rescuing apoptosis. This study showed that FKBP2 is anti-apoptotic, negating the toxic effects of human Bax protein. This connects the protein folding pathway in apoptotic processes. It is known that some toxic proteins usually aggregate before they exert their toxicity on cells. There is a strong argument for the involvement of proteins in the protein folding pathway, which could prevent aggregation. This sort of action could be important to the survival of cells and tissues where the aggregation occurs, such as neurodegenerative diseases. Further studies to elucidate the pathways involved will be of vital importance. FKBP2 possibly acts as a molecular chaperone that prevents aggregation and misfolding of proteins in the endoplasmic reticulum, reducing proteotoxic stress. This study suggests that FKBP2 play a cytoprotective role when co-expressed with Bax protein in yeast cells. However, the mechanism underlying this anti-apoptotic characteristic of FKBP13 remains largely unknown and needed to be explored further.

## Supplementary Information

Below is the link to the electronic supplementary material.

Supplementary file1 (DOCX 47 KB)

## Data Availability

All data generated or analysed during this study are included in this published article [and its supplementary information files].
