# Supplementary material for: FK506-binding protein 2 (FKBP13) inhibit Bax-induced apoptosis in Saccharomyces cerevisiae (yeast)
Source: Cell Biol Toxicol. 2021 Aug 3;39(3):719–28. doi: 10.1007/s10565-021-09633-w (PMC10406727; doi:10.1007/s10565-021-09633-w)
Supplement: Supplementary file 1 — Supplementary file1 (DOCX 47 KB) [file 10565_2021_9633_MOESM1_ESM.docx]

**Supporting information**

**FK506-binding protein 2 (FKBP2) inhibit Bax induced apoptosis in Saccharomyces cerevisiae (Yeast)**

Damilare D. Akintade^a,b^* and Bhabatosh Chaudhuri^b^

^a^ School of Clinical and Applied Sciences, Leeds Beckett University. LS1 3HE, UK

^b^ Leicester School of Pharmacy, De Montfort University, Leicester, LE1 9BH, UK

* Corresponding author

Dr Damilare Akintade

E-mail: D.Akintade@leedsbeckett.ac.uk

^a^ Current address: School of Clinical and Applied Sciences, Leeds Beckett University. LS1 3HE, UK

**Contents**

Section 1. The plasmid construct bearing the human Bax-α gene and the resultant yeast strain.

Section 2. Plasmid constructs bearing the human FK506 binding protein (FKBP2) gene and the resultant yeast strains.

Section 3. Plasmid constructs bearing the Bcl-xL gene and the resultant yeast strains.

**SECTION 1**: **The plasmid construct bearing the human Bax-α gene and the resultant yeast strain**

The human *Bax-α* (*Bax*) gene is under the control of the galactose-inducible *GAL1* promoter and was cloned in an integrating vector that bears the *LEU2* auxotrophic marker. The resultant plasmid was integrated into the *LEU2* chromosomal locus of the yeast strain W303 1A1. The map of the plasmid is shown below.

**Figure 1**. The plasmid map of pRS305/GAL1p-h_Bax-MS showing restriction sites that cut the plasmid only once. The human Bax gene contains, at the 3’-end, a sequence that codes for the *c*-myc tag so that expressed protein can be monitored with an antibody that recognizes the *c*-myc epitope at the C-terminus of Bax protein.

**SECTION 2**: **Plasmid constructs bearing the human FK506 binding protein (FKBP2) gene and the resultant yeast strains**

The human FKBP2 gene was cloned downstream of the PGK1 promoter in episomal plasmids bearing URA3 auxotrophic marker. The plasmids were then transformed into the yeast strains W303 1A1 carrying one copy of Bax gene. The plasmid map is shown below.

**Figure 2.** Plasmid map of pSYE239/FKBP2-HA, an episomal 2u-plasmid encoding HA-tagged FKBP2 gene. The restriction sites shown are the ones that cut the plasmid only once.

**SECTION 3**: **Plasmid constructs bearing the Bcl-xL gene and the resultant yeast strains**

The Bcl-xL gene was cloned downstream of the PGK1 promoter in episomal plasmids bearing URA3 auxotrophic marker. The plasmids were then transformed into the yeast strains W303 1A1 carrying one copy of Bax gene. The plasmid map is shown below.

**Figure 3.** Plasmid map of pSYE239/Bcl-xL-HA, an episomal 2u-plasmid encoding HA-tagged Bcl-xL gene. The restriction sites shown are the ones that cut the plasmid only once.
